# Effect of Oxidized Low Density Lipoprotein on the Expression of *Runx2* and *SPARC* Genes in Vascular Smooth Muscle Cells

**DOI:** 10.7508/ibj.2015.03.005

**Published:** 2015-07

**Authors:** Effat Farrokhi, Keihan Ghatreh Samani, Morteza Hashemzadeh, Mohammad Amin Tabatabaiefar

**Affiliations:** 1 *Cellular and Molecular Research Center, Shahrekord University of Medical Sciences, Shahrekord, Iran; *; 2 *Clinical Biochemistry Research Center, Shahrekord University of Medical Sciences, Shahrekord, Iran;*; 3 *Dept. of Genetics and Molecular Biology, School of Medicine, Isfahan University of Medical Sciences, Isfahan, Iran*

**Keywords:** Vascular calcification, Oxidized low density lipoprotein, Osteonectin, Runx2

## Abstract

**Background:**

Vascular calcification is an important stage in atherosclerosis. During this stage, vascular smooth muscle cells (VSMC) synthesize many osteogenic factors such as osteonectin (encoded by *SPARC)*. Oxidative stress plays a critical role in atherosclerosis progression, and its accumulation in the vascular wall stimulates the development of atherosclerosis and vascular calcification. The osteonectin overexpression has been observed in the arterial wall during the course of atherosclerosis. However, the regulatory mechanism of oxidized low density lipoprotein (oxLDL)-mediated vascular calcification remains to be clarified. The aim of this study was to investigate the effect of oxLDL on the osteonectin gene expression through the Runx2 transcription factor.

**Methods:**

In this experimental study, VSMC were cultured in F-12K media and then treated with oxLDL. The expression of Runx2 and osteonectin genes was determined by real-time PCR method. Protein levels were investigated by the western blotting technique. The Runx2 gene was knocked down using siRNA in order to determine whether Runx2 regulates the osteonectin expression in VSMC induced by oxLDL. Then transfected cells were treated with oxLDL, and the expression levels of Runx2 and osteonectin were determined again.

**Results:**

oxLDL was found to increase Runx2 and osteonectin gene expression (4.8 ± 0.47- and 9.2 ± 1.96-fold, respectively) after 48 h. Western blotting analysis confirmed the induced levels of Runx2 and osteonectin proteins. However, oxLDL-induced osteonectin expression was not observed to be blocked by Runx2 knockdown.

**Conclusion:**

The up-regulation of osteonectin by oxLDL is independent of Runx2, and it may be mediated by other transcription factors.

## INTRODUCTION

Vascular calcification, defined as the pathologic deposition of mineral in the vascular system, is generally observed in atherosclerosis [1]. Vascular calcification is widely believed to be an active process similar to bone formation. In this regard, bone-related proteins, such as osteonectin and osteocalcin, as well as bone morphogenic proteins have been reported in calcified vascular tissues [2, 3]. 

Osteonectin, which is known as secreted protein acidic and rich in cysteine (*SPARC*), is a bone-related protein that has a major role in bone development and mineralization. It has high affinity for hydroxyapatite and calcium [4]. Like other bone-related proteins, osteonectin is expressed in the arterial wall during atherosclerosis progress, specifically during the calcification of the atherosclerotic plaque [3, 5, 6]. The presence of this protein in advanced human atherosclerotic plaques suggests that osteonectin may act as a promoter of vascular calcification [6]. The *osteonectin* gene is strongly expressed in aortic stenosis and participates in the neovascularization of aortic stenosis valves [7]. This protein also plays an important role in atherogenesis and can serve as a new biomarker of atherosclerosis and calcinosis of coronary arteries [8].

Oxidative stress is a vital factor in the progression of vascular calcification [9] and activates the genes that lead to the increased deposition of extracellular matrix proteins [10]. Oxidized low-density lipoprotein (oxLDL) accumulation in the vascular wall stimulates the development of atherosclerosis and vascular calcification [11]. 

Runx2 is a key transcription factor necessary for osteoblast differentiation and regulation of the expression of many osteogenic factors [12]. *In vitro* studies have demonstrated that Runx2 has an essential role in oxidative stress-induced vascular smooth muscle cells (VSMC) calcification, and Runx2 alone is sufficient to induce VSMC calcification [12,13]. However, the potential link between the *osteonectin* gene expression and oxidative stress-induced vascular calcification has not been examined. The current study was launched to investigate the hypothesis that oxLDL up-regulates *osteonectin* expression in human VSMC through Runx2.

## MATERIALS AND METHODS

The human aorta VSMC and F12K media were purchased from Pasteur Institute of Iran (Tehran), OxLDL from Biomedical Technologies (Stoughton, MA, USA). cDNA synthesis kit, DNase, and SYBR Green PCR Master Mix were obtained from Thermo Fisher Scientific (Waltham, MA, USA), Trizol from Invitrogen (USA), siRNA (S2455,S2456), Opti-MEM media, and lipofectamine from Invitrogen (Ambion, Austin, TX, USA), FITC (fluorescein isothiocyanate)-conjugated siRNA from Santa Cruz Biotechnology (USA), anti-Runx2 (ab102711), anti-SPARC (ab55847), anti-beta-actin (ab70165), and secondary antibody (ab6721) from Abcam (Cambridge, UK), and 3, 3’-5, 5’-tetramethylbenzidine (BM blue) from Roche Diagnostic (Mannheim, Germany).

***Cell culture, RNA isolation, and cDNA synthesis. ***In this experimental study, human aorta VSMC were cultured in F-12K media containing 10% fetal bovine serum. The media contained 0.05 mg/mL ascorbic acid, 0.01 mg/mL insulin, 0.01 mg/mL transferrin, 10 ng/mL sodium selenite, 0.03 mg/mL endothelial cell growth supplement, 10 mM HEPES, 10 mM TES, 100 U/mL penicillin, 100 µg/mL streptomycin, and 0.01% amphotericin B. The cells were incubated at 37°C in a humidified atmosphere containing 5% CO_2_. Cell growth was followed daily, and cells from passages 3-7 were used for all experiments. Cells were cultured with 10 mM β-glycerophosphate for 12 days to induce VSMC calcification, and then they were seeded in 12-well plates at an initial density of 10,000 cells per well. Having being achieved approximately 80% confluence, the cells were transferred to media containing 100 µg/mL oxLDL [14]. The cells cultured in the media containing β-glycerophosphate without oxLDL were used as the control. Total RNA was isolated using Trizol according to the manufacturer's instructions after 24 and 48 h. RNA was quantified using a NanoDrop 2000C spectrophotometer (Thermo Scientific, USA) and treated with DNase. Then cDNA was synthesized from 0.5 µg total RNA using a random primer and the ReverAid First Standard cDNA Synthesis kit (Thermo Scientific, Canada).

***Real-time PCR. ***Quantitative real-time PCR was performed using the Rotor-Gene 3000 real-time DNA amplification system (Corbett Research, Australia) and SYBR green method. The sequences of primers used for real-time PCR are listed in Table 1. The amplification was carried out as follows: initial enzyme activation at 94°C for 10 min, then 40 cycles of 95°C for 15 s, 59°C for 20 s, and 72°C for 30 s. The quantitation of data was performed using the comparative C_T_ (ΔΔC_T_) method by *glyceraldehyde-3-phosphate dehydrogenase* gene expression as an endogenous reference.

***Western blotting.*** After appropriate treatment, VSMC were washed twice with cold PBS and lysed in ice-cold radio immune precipitation assay buffer (6×) containing protease inhibitor cocktail. The homogenate was incubated in lysis buffer for 30 min and then centrifuged at 12,000 ×g at 4°C for 10 min. The supernatant was used as the total cell lysate. Protein concentration was measured spectrophoto-metrically by a NanoDrop, and the equal amounts of protein from each sample were subjected to blotting. Protein lysate was mixed with SDS loading buffer (0.125 M Tris-HCl 4%, SDS 20%, glycine 10%, and 2-mercapto-ethanol), followed by boiling for 5 min and separated by 12% SDS-PAGE. The separated proteins were then transferred to polyvinylidene difluoride membranes in Tris-Glycine buffer (25mM Tris-base, 192 mM glycine, 20% methanol) for 2 h at 120 V. The membranes were blocked using blocking solution (5% nonfat dried milk in Tris-buffered saline and 0.1% Tween-20) at 4ºC overnight. Then they were incubated in Tween-20 containing the primary rabbit polyclonal anti-SPARC (1 μg/mL) and primary rabbit polyclonal anti-Runx2 (1:2,000) at room temperature for 2 h. After washing, the membrane was incubated with goat anti-rabbit IgG horseradish peroxidase conjugate (1:10,000) at room temperature for 90 min. Finally, the color was developed with the addition of 3,3’,5,5’-tetramethyl-benzidine membrane peroxidase substrate. The color reaction was stopped by washing the membranes with distilled water. Cell lysates were detected on a separate membrane with actin as a loading control. 

**Table 1:** **.** Primer sequences and product lengths

**Genes**	**Primer sequences (5'–3')**	**Product length (bp)**	
*Runx2*	F: CGATCTGAGATTTGTGGGCC R: GGGAGGATTTGTGAAGACGG		76
*Osteonectin*	F: TCTTCCCTGTACACTGGCAGTTC R: AGCTCGGTGTGGGAGAGGTA		73
*GAPDH*	F: ACACCCACTCCTCCACCTTTG R: TCCACCACCCTGTTGCTGTAG		112

GAPDH, glyceraldehyde-3-phosphate dehydrogenase; F, forward; R reverse

***Runx2 RNA interference. ***To knockdown *Runx2*, we used two siRNA (ID: s2455 and s2456). Runx2 siRNA, s2455 and s2456, are located in the region of the Runx2 transcript that codes for amino acids 910-928 and 1663-1683, respectively. The oligonucleotide sequences were as follows: s2455: 5'-CUUGAUGAC UCUAAACCUATT-3' and 5'-UAGGUUUAGAGUC AUCAAGCT-3', s2456: 5'-CCAAAUUUGCCUA ACCAGATT-3' and 5'- UCUGGUUAGGCAAAUUU GGAT-3'. The cells were seeded in a 24-well plate at a density of 20,000 cells/well in growth media without antibiotics. After 24 h, the cells were transfected with RNAi duplex-Lipofectamine RNAiMAX complexes made in Opti-MEM according to the manufacturer’s instructions. siRNA (10 nM each) was used for all transfections. siRNA transfection efficiency was observed by the uptake of FITC-labeled siRNA sequence and scramble oligoribonucleotide duplex that was not homologous to any mammalian genes was used as a control. After 24 h, the transfection media were removed, and the cells were incubated for an additional 24 h in normal growth media and then treated with oxLDL (100 µg/mL). The cells were harvested for mRNA and protein extraction after 48 h.

***Statistical analysis. ***Data were  presented as the mean ± SEM of three independent experiments. Statistical analysis was carried out using nonparametric Kruskal-Wallis test, and pairwise comparisons between the groups were performed by Mann-Whitney test. All statistical analyses were performed by Graph Pad Prism5 software v5.01, and all data were presented as mean ± SEM, and *P* < 0.05 was regarded as the level of significance. 

## RESULTS

**Fig. 1.**
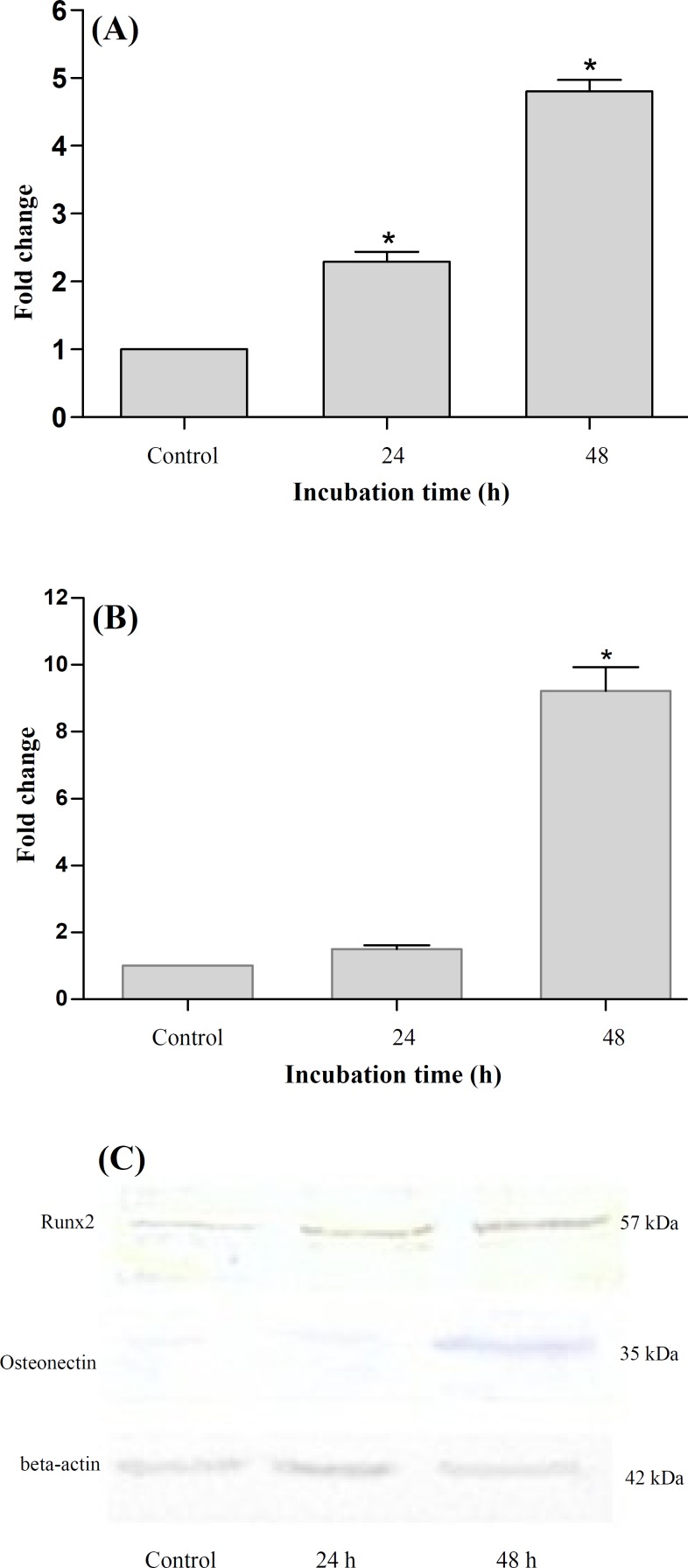
The up-regulation of Runx2 and osteonectin expression in VSMC by oxLDL. The cells were treated by oxLDL for 24 and 48 h. The expressions of Runx2 (A) and osteonectin (B) against control were determined by real-time PCR after treatment. Data were expressed as means ± SEM of three independent experiments. ^*^P < 0.05 compared with control; (C) Protein expression levels of Runx2 and osteonectin were determined by western blotting analysis against control (beta-actin

**Fig. 2:**
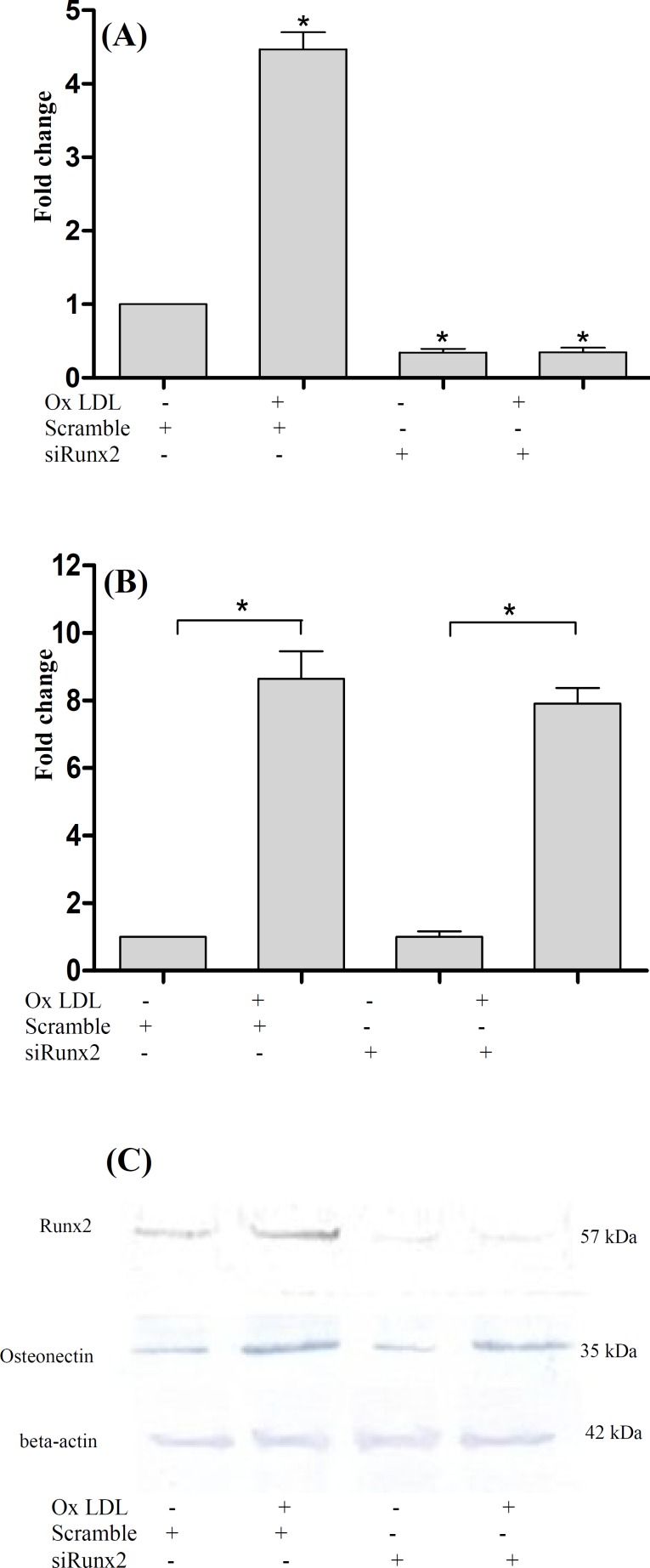
A decrease in Runx2 expression by the Runx2 knockdown. After the transfection of cells with Runx2 siRNA or control siRNA (scramble), the cells were treated with or without oxLDL (100 µg/mL) for 48 h. Then real-time PCR (A and B) or western blotting analysis (C) was performed. Data were expressed as means ± SEM of three experiments. ^*^P < 0.05 was compared with contro

***Effect of oxLDL on*** ***osteonectin and Runx2 expression. ***VSMC were treated with 100 µg/mL oxLDL to investigate the effect of oxLDL on *Runx2* and *osteonectin* expression. The expression of osteonectin and Runx2 in mRNA and protein levels were analyzed using real-time PCR and western blotting, respectively. Our result indicated that oxLDL increased Runx2 expression (2.29 ± 0.39- and 4.8 ± 0.47-fold) and osteonectin expression (1.49 ± 0.35- and 9.2 ± 1.96-fold) after 24 and 48 h, respectively (Fig. 1). siRNA transfection. To determine whether Runx2 knockdown may affect oxLDL-induced expression of *osteonectin*, we compared the expression of the *osteonectin* gene in control and Runx2-knockdown cells. First, the cells were transfected with Runx2 treated with oxLDL. Transfection efficacy was determined using FITC-labeled siRNA and fluorescence microscopy. The transfection efficiency was > 70% as determined by fluorescence. Real-time PCR and western blotting results showed that Runx2 siRNA clearly suppressed both basal and oxLDL-induced Runx2 expression (Fig. 2A and 2C). However, oxLDL-induced osteonectin expression was not blocked by Runx2 knockdown (Fig. 2B and 2C). These results indicated that oxLDL-induced *osteonectin* expression was not dependent on Runx2 expression, suggesting that up-regulation of *osteonectin* by oxLDL is independent of Runx2, and it may be mediated by other transcription factor.

## DISCUSSION

In this study, we showed that oxLDL increased the expression of *Runx2* and *osteonectin* genes in VSMC, and silencing of *Runx2* had no effect on the expression of the osteonectin gene. 

Vascular calcification is a process actively regulated by osteogenic factors [15, 16]. However, the mechanisms underlying the up-regulation of these factors have not been fully understood. Osteonectin is an osteogenic marker, and the presence of this protein in advanced human atherosclerotic plaques has been already described [6]. We found that the stimulation of VSMC with oxLDL increased osteonectin protein levels. This observation indicates that oxLDL-induced osteonectin expression may induce vascular osteogenic changes associated with atherosclerosis. It is known that oxLDL accumulation in the vascular wall prompts the development of atherosclerosis and vascular calcification [11] and based on our finding, oxLDL contributes to the development of vascular calcification by the up-regulation of osteogenic factors such as osteonectin. 

Another result of this study was that oxLDL increased the expression of *Runx2*, which is a transcription factor with a central role in osteoblast differentiation and bone formation. Runx2 is a pivotal player in VSMC calcification *in vitro* [13, 17], and its elevated protein levels in atherosclerotic plaque also suggests its possible main role in vascular calcification [18].

A recent study has demonstrated that Runx2 deficiency eliminates the oxidative stress-induced expression of osteogenic markers such as alkaline phosphatase and osteocalcin [19]. Also, another study has indicated that Runx2 suppression reduces the transcription of osteoblast markers such as osteonectin, osteocalcin, and alkaline phosphatase [20]. Although we had hypothesized that oxLDL would increase the osteonectin expression by up-regulating Runx2 expression, our findings showed otherwise; Runx2 did not mediate oxLDL effect on osteonectin expression.

Therefore, other factors should mediate the effect of oxLDL on the *expression* of osteonectin. Osterix is another osteoblast transcription factor involved in the regulation of numerous osteoblast genes, including osteocalcin, osteonectin, osteopontin, bone sialo-protein, and collagen type I [21]. 

Since oxLDL promotes osteoblast differentiation in VSMC by up-regulating osterix expression [14], it seems that other regulators such as osterix which mediates the effect of oxLDL on *osteonectin* expression. However, future studies are needed to confirm the hypothesis.

In summary, the findings of this study demonstrated that oxLDL increased the expression of Runx2 and osteonectin genes in VSMC. Neverthless, oxLDL-induced osteonectin expression was not blocked, and it may be mediated by other transcription factor such as osterix.
